# TRAIL Conjugated Silver Nanoparticle Synthesis, Characterization and Therapeutic Effects on HT-29 Colon Cancer Cells

**DOI:** 10.22037/ijpr.2020.112069.13514

**Published:** 2021

**Authors:** Fatih Birtekocak, Gulen Melike Demirbolat, Ozge Cevik

**Affiliations:** a *Department of Biochemistry, School of Medicine, Aydin Adnan Menderes University, Aydin, Turkey. *; b *Department of Pharmaceutical Technology, Faculty of Pharmacy, Biruni University, Istanbul, Turkey.*

**Keywords:** TRAIL, Colon Cancer, Silver Nanoparticles, Apoptosis, HT-29 cells

## Abstract

Colon cancer is one of the most prominent causes of cancer-related morbidity and mortality and curable if detected in the early stages. TNF-related apoptosis-inducing ligand (TRAIL) is a therapeutic protein and has a potential anti-cancer activity that is widely used for the treatment of several cancers. In this study, we aimed to develop a silver nanoparticle system conjugated with TRAIL and coated with PEG (AgCTP NPs) to improve the therapeutic effects of colon cancer. AgCTP NPs were characterized by UV spectrum, FTIR and zetasizer. Cytotoxicity, hemolysis assay and apoptotic effects of nanoparticles were investigated using a colon cancer cell line (HT-29) *in-vitro*. Treatment with AgCTP NPs effectively inhibited proliferation and colony formation of HT-29 cells. The apoptotic effects of nanoparticles on HT-29 cells were determined as Bax, Bcl-2, PARP and clv-PARP protein expression levels using Western blot. Apoptotic proteins were upregulated by AgCTP NPs. In this study, we demonstrated that AgCTP NPs had an anti-cancer effect by activating cell death. Thus, we have confirmed that silver nanoparticles can be selected as a good carrier for TRAIL therapeutic proteins that can be used to treat colon cancer.

## Introduction

Colorectal cancer, commonly called colon cancer, is the third most widely diagnosed cancer worldwide and is highly preventable and curable if detected in the early stages (1, 2). There are many different colon cancer therapies, including surgery, chemotherapy and radiation therapy which is a complementary therapy. However, these therapies are usually inefficient due to non-effective anti-cancer agents in targeting sites (3). Because higher doses can also lead to adverse effects, it should not be used. In addition to cancerous cells, healthy cells are also damaged in these processes. Therefore, alternative systems should be developed for effective dose delivery.

Nanotechnology, a multidisciplinary field covering many fields such as physics, chemistry, biology, engineering and medicine, has recently emerged as one of the most favorable areas for cancer treatment (4). It has an incredible potential to revolutionize drug delivery and delivery systems that represent the optimal application of nanoparticles (NPs) (5). Considering the chelating ability and workability of metal NPs, an effective dose amount can be delivered to the target site after therapeutic agents are loaded onto the NPs (6). Additionally, NPs tend to accumulate more in tumor tissue than in normal tissues via the enhanced permeability and retention (EPR) effect and can efficiently be taken up by the cells (7-9). Employing rationality of the EPR effect is that the NPs cannot pass through the openings of normal vessels, which are less than 10 nm in size and would enter the openings of the tumor vessels, which can be hundreds of nanometers (10). Therefore, NPs fascinate many researchers for drug delivery systems. Especially, gold and silver NPs have recently been used as drug carriers (11, 12). Silver NPs can also be used in medical imaging, biosensors, nanocomposites, antimicrobials and cell electrodes (13). It is known that silver has better light absorption compared to other metal ions so that better resolution and better affinity can be provided for functionality (14, 15).

TNF-related-apoptosis-inducing ligand (TRAIL) is a member of the tumor necrosis factor (TNF) superfamily (16). It was first cloned and identified in 1995 by Wiley *et al. *(17). TRAIL protein (theoretically about 23 kDa) contains 281 and 289 amino acid residues in the human and murine forms, respectively, both sharing 65% sequence identity (18). It is expressed on the surface of natural killer (NK), T cells, macrophages and dendritic cells (19). TRAIL can favorably induce apoptosis in various primary tumor cell lines such as colon, breast, lung, skin, thyroid, prostate, kidney, pancreas and central nervous system (20) through interacting with its proapoptotic death receptors (DR4 and DR5) (20, 21) but not in normal cells. Therefore, it has attracted a number of research laboratories and pharmaceutical companies, and they have desired to develop recombinant forms of TRAIL (rTRAIL) or TRAIL receptor agonists for therapeutic purposes (22).

TRAIL can induce apoptosis up to 5 ng/mL, but its biological half-life is quite short in rTRAIL (23-25). Namely, TRAIL has poor stability. Therefore, the effective dose amount of TRAIL cannot reach the tumor tissue (26). This disadvantage can be overcome by NPs having stability and EPR effect and/or through coating processes such as PEGylation, thereby enabling higher and effective dose delivery to the target site. 

When NPs enter the body, they can inevitably interact with biological molecules such as proteins to form the Corona or replace the existing ligand with other molecules (27). Therefore, NPs seem reasonable to cover with a coating material such as PEG. Additionally, it is widely reported that the PEGylated NPs have a “secret” character that can delay or prevent by the rapid cleaning of the reticuloendothelial system (RES) (28, 29). Furthermore, PEGylation improves the targeting of cancerous cells through the EPR effect (30).

## Experimental

*Materials And Methods*

*Design, Production, and Characterization of rTRAIL protein*

TRAIL gene (Ensemble: ENSG00000121858) was amplified from human TRAIL cDNA. XhoI and Notl restrictions sites had been added at the 5’end of the forward and reverse primers, respectively, and their sequences were as follows: 5`ATGC*CTCGAG*A TAGAGAAGGAAGGGCTTCAGTG`3 and 5`ATGC*GCGGCCGC* TGCGATCTTTTAGTGGTGCCT `3.

 Primer was designed and analyzed with software (Primer-BLAST, NCBI). The amplification of the human TRAIL gene was performed using DNA Polymerases (F530S Phusion High-Fidelity DNA Polymerases, Thermo Scientific) with PCR (T100 Thermal Cycler, Biorad). pET28a, his tagged bacterial expression plasmid (EMD Biosciences, USA), was digested with XhoI and Notl (R0146S and R0189S, New England Biolabs). Then TRAIL gene was ligated to pET28a and named pET28a.TRAIL. The pET28a.TRAIL expression plasmid was also transformed into *Escherichia Coli* (*E. coli)* BL21 (DE3). *E. coli* was cultured in LB with Kanamycin (Santa Cruz, USA) at 100 µg/mL, and recombinant protein expression was induced with 1 mM isopropyl β-d-1-thiogalactopyranoside (IPTG, Sigma). Recombinant TRAIL protein (rTRAIL) was purified on Ni-NTA affinity beads (Santa Cruz). Protein was controlled with 15% SDS-PAGE gels and staining Coomassie brilliant blue. Commercial TRAIL protein (Sigma T9701) was used as a positive control to TRAIL purification. 

*Synthesis of silver nanoparticles*

After the magnetic stirrer was adjusted to 37 °C, 100 mL of met-NaOH (0.02 M, 99%) solution and 1 mL of cysteine solution (0.1 M) were added in a 250 mL beaker. pH was adjusted to 9 by 1M HCl and 2N NaOH, and then 1 mL of AgNO_3_ (0.1 M) solution was added dropwise to this mixture. One-hundred μL TRAIL (20 μg/mL) and 100 μL PEG_400_ were respectively added in the same way after every 10 min and stirred for 4 h. The mixture was centrifuged for 5 min at 4000 rpm (Hettich 320R, Germany). To remove impurities, the pellet was separately washed 3 times with methanol and dH_2_O. The powder form of nanoparticles obtained by lyophilization and was kept at 4 °C for further studies. AgC (Silver-cysteine), AgCT (Silver-cysteine-TRAIL) and AgCTP (Silver-cysteine-TRAIL-PEG) NPs were synthesized by the same procedure.

*Characterization of silver nanoparticles*

Polarized light-absorbing properties of AgCT NPs were examined by Ultraviolet-visible (UV-Vis) spectrophotometer (Thermo Scientific Multiskan Spectrum 1500, USA) with a range of 200-800 nm. The functional groups of AgCT NPs were confirmed by Fourier-transform infrared (FTIR) spectroscopy in the range of 650-4000 cm^-1 ^(PerkinElmer UATR two, U.S.). Particle size and zeta potential values of the synthesized AgCT NPs were determined by Zetasizer (Nano ZS-90 Malvern Instruments, England). 

*Cell culture and cell survival assay*

HT-29 human colon cancer cell lines were purchased from the American Type Culture Collection (ATCC, Manassas, VA, USA). These cells were cultured in Dulbecco’s Modified Eagle’s Medium (DMEM) supplemented with 10% FBS, 2 mM L-glutamine, 100 U/mL penicillin and 100 μg/mL streptomycin, and kept at 37 °C under 5% CO_2_. To determine the cell viability, cells were trypsinized and seeded into 96-well plates (1 × 10^4^ cells/well). The cells were treated with different concentrations (1.56–25 ng/mL) of AgCT NPs. Control cells were incubated without AgCT NPs. All wells were incubated for 24 h, then washed with PBS and added to 100 μL DMEM. For MTT assay, ten microliter of the MTT (3-(4,5-dimethylthiazol-2-yl)-2,5-diphenyltetrazolium bromide) (Vybrant, Invitrogen) labeling reagent was added to this wells and incubated for 4 h in a humidified atmosphere at 37 °C incubators with 5% CO_2_ in the air. After the incubation, 100 μL of the SDS buffer was added into each well for solubilization of formazan precipitate. Then absorbance was measured by microplate reader at 570 nm (Thermo Scientific Multiskan Spectrum 1500, USA). Each analysis was repeated 3 times. Cell viability was calculated as follows:

Cell viability% = (Abs_(sample) _- Abs_(blank)_)/(Abs_(control) _- Abs_(blank)_) *×* 100

*Cell growing assay*

In order to investigate the effects of AgCT NPs on cell survival of HT-29 cells, these cells were treated with AgCT NPs, a final concentration of 1.19 ng/mL, in DMEM without antibiotics. In microscopic analysis, images were collected under an inverted microscope (Olympus CX51) with bright light. After 24 h, methylene blue staining was used in cell viability for Microscopic analysis. The cells extracted with 1% SDS in PBS solution were stained with 0.01% methylene blue solution and then spectrophotometrically was evaluated at 600 nm. Each experiment was repeated three times.

*Colony formation assay*

For evaluating colony formation, HT-29 cells seeded in six-well plates (1000 cells per well) were treated with AgCT NPs and cultured in a growth medium for 14 days. The medium was refreshed every 3 days until incubation was completed. At the end of incubation, each well in the plate was washed with PBS, fixed with cold methanol/acetic acid, stained with 0.5% crystal violet staining solution for 15 min and washed with dH_2_O, respectively. The stained cells were examined with a microscope. The number of colonies in each well was counted and analyzed.

*Hemolysis Assay*

The freshly obtained blood sample was put into tubes involving EDTA and centrifuged at 1000 ×*g* for 15 min. After discarding the supernatant, 10% (w/v) RBC solution was prepared with pellet in PBS (pH 7.4). One-hundred μL RBC solution and 900 μL AgCT NPs with different concentrations (25, 12.5 and 6.25 ng/mL) were mixed in a microtube and then were incubated at 37 °C for 1 h. By the way, 900 μL 0.9% NaCl and dH_2_O were used for negative and positive control, respectively. The mixtures were centrifuged at 1000 ×g for 15 min following incubation and supernatants were spectrophotometrically measured at 540 nm. The experiments were repeated three times. The percentage of hemolysis was calculated as follows:

Hemolysis% = (Abs_(sample) _- Abs _(-control)_)/(Abs _(+control) _- Abs_ (-control)_) *×* 100

According to this formula, the negative control was accepted as blank.

*Western blot analysis*

HT-29 cells were lysed with RIPA buffer after incubation with AgCT NPs. Protein detection was performed with bicinchoninic acid assay (BCA) and then 30 µg protein was loaded per well in 12% SDS-PAGE gel. Proteins were separated by SDS-PAGE and transferred onto PVDF membrane (sc-3723, Santa Cruz). The membrane was blocked with 3% BSA for 2 h at room temperature and then was incubated with primary antibodies (1:500 dilution of Bax, Bcl-2, PARP, clv-PARP and β-actin from Santa Cruz) at 4 °C overnight. After the incubation with primary antibody, PVDF membrane was washed with TBST (Tris-buffered saline, 0.1% Tween 20) and incubated with horseradish peroxidase-conjugated secondary antibody (mouse anti-rabbit IgG-HRP and goat anti-mouse IgG_1_-HRP from Santa Cruz) for 2 h at room temperature. Then the membrane was washed with TBST. Bands were visualized by the chemiluminescence Western blotting detection reagent (sc-2048, Santa Cruz) in imaging system (G:BOX Chemi XRQ SynGene, USA). β-Actin protein levels were used as a control to equal loading of the gel. The experiments were repeated three times.

*Statistical analysis*

The student *t*-test and analyses of variance (ANOVA) test were used for statistical analysis. If the *p*-value which is used to determine the level of statistical significance and difference, was equal to 0.05 or less than 0.05, it was considered significant. All data were represented as mean ± SD unless otherwise indicated. All experiments were repeated three times.

## Results

*Synthesis of Silver Nanoparticles*

**Figure 1:**
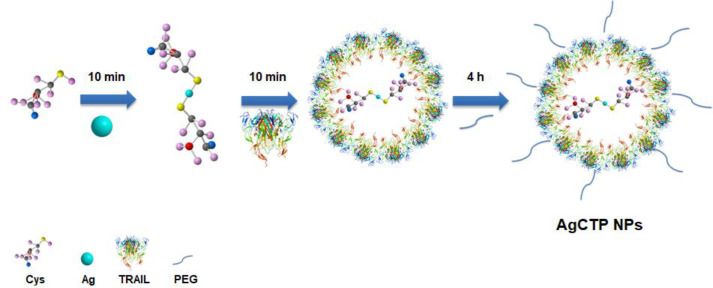
Schematic representation of synthesis AgCTP nanoparticles

The purpose of the presented studies was to obtain TRAIL conjugated silver nanoparticles dedicated for colon cancer therapy. Because the sulfur end of cysteine was negatively charged due to the deprotonation of sulfur moiety, pH was chosen as 9.00. Thus, the synthesized AgC molecules were bounded with an Ag-S chemical bond. However, AgC molecules were coated with TRAIL to form AgCT molecules by hydrogen bonds. PEG molecules in AgCTP NPs were also bonded to AgCT NPs by a hydrogen bond. Figure 1 shows the synthesis steps of AgCTP NPs.

*Characterization of synthesized AgNPs*

Normally, silver(I) nitrate react with sodium hydroxide to produce silver(I) oxide by the reaction: 2AgNO_3_ + 2NaOH → Ag_2_O + 2NaNO_3_ + H_2_O 

Silver oxide is a black or dark brown color and slightly soluble in water. However, the AgCT NPs which we synthesized were light brown and had good water solubility. Based on this informations and our previous experiences, we are confident that synthesized AgCT NPs were different molecules from Ag_2_O.

The color change is usually occurring in the synthesis of metal NPs, so visual observation is a good sign of metal NPs synthesis. During the synthesis, this change is caused by the excitation of the surface plasmon resonance (SPR) effect on metal NPs. Our experiments showed that the appearance of a light brown color was evidence of the formation of the AgNPs. In addition to quantitative results, we also performed qualitative assessments to confirm the synthesis of AgNPs. The supernatant and pellet for AgC binding control were subjected to a modified ninhydrin test. The modified ninhydrin test is specific to amino acids (31). Namely, this test can be used to verify the absence or presence of amino acids. The pellet had a purple-red color for the ninhydrin test, while the supernatant had a clear color (Supplementary file, Figure S1a). The result of this test was positive only for pellet. According to the ninhydrin experiment results for pellet of AgC NPs, Ag and cysteine were in the same environment. However, the main problem was whether they were bonded by the Ag-S chemical bonding. Therefore, we used the nitroprusside test. The nitroprusside test is positive for the presence of free -SH group, but it was negative for pellet of AgC NPs (Supplementary file, Figure S1b). Obviously, the free -SH end in the cysteine lost proton and interacted with Ag to form the Ag-S chemical bonding. 

In this study, TRAIL expression by *E. coli* BL21(DE3) was generated by pET28a.TRAIL vector. The pET28a.TRAIL vector is predicted to encode a recombinant protein with a molecular size of 24 kDa in the induction of IPTG (Supplementary file, Figure S1c). To check whether TRAIL binds to AgC NPs, the Bradford test was used in the AgCT NPs. The Bradford test is one of the methods used to verify the presence of proteins and to calculate protein quantity up to 1 microgram. The test result for AgCT NPs was positive in both pellet and supernatant of AgCT (Supplementary file, Figure S1d). However, in our preliminary trials, only the supernatant of AgCTP was negative. The pellet for AgCT NPs had dark blue color, but the supernatant had a light blue. Additionally, OD_595_ values were 0.089 ± 0.012 (AgCT pellet) and 0.023 ± 0.007 (AgCT supernatant). When the standard chart was drawn against BSA, the amount of free TRAIL found about 0.2 μg. It was quite normal because some of TRAIL might have released from AgCT NPs due to centrifugal forces, and/or some of the TRAIL might not have bounded to AgC NPs. According to the gel electrophoresis results, we could not observe a single TRAIL band other than AgCT and AgCTP NPs bands (Supplementary file, Figure S1e). This means that free TRAIL molecules were removed from AgCT and AgCTP NPs as much as possible during the washing of Ag NPs pellet. In addition, the presence of TRAIL bands of AgCT and AgCTP molecules at different locations in gel electrophoresis indicates that the AgCTP molecule was correctly pegylated.

**Figure 2:**
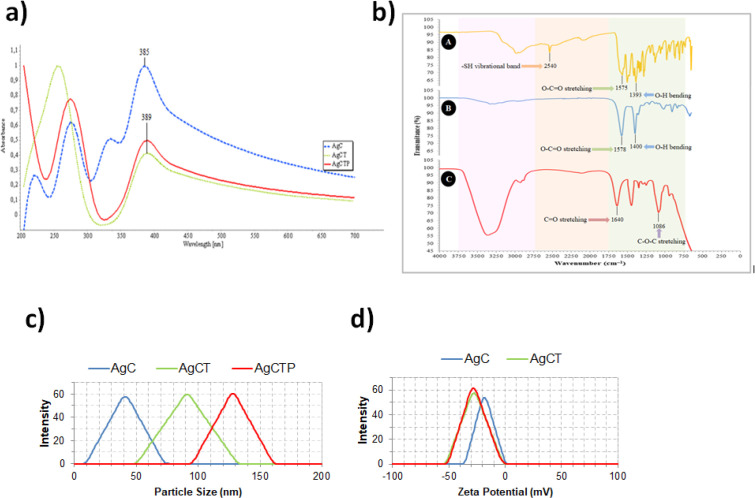
Characterization of AgCT NPs conjugated with TRAIL, (a) UV-Vis spectrum of AgNPs, (b) FTIR spectrum of cysteine (A), AgC (B) and AgCTP (C) molecules FT-IR spectrum (c), particle characterization, (d) zeta potential

Figure 2a shows UV-Vis spectra from different AgNPs. These spectrums indicated the formation of AgC, AgCT and AgCTP NPs, which were different from each other. The absorption spectrum of AgCT and AgCTP NPs spanned a wide range from 200 to 700 nm with a prominent peak at 389. However, AgC NPs had a prominent peak at 385. 

The prominent –SH vibrational band at 2540 cm^-1^ in the FTIR spectrum was clearly appeared in the free cysteine molecule but not in the AgC and AgCTP (Figure 2b). The lack of the band at 2540 cm^-1^ showed the formation of Ag–S covalent bond in the AgC and AgCTP NPs (32, 33). According to the literature data, the characteristic vibrational band of Ag-S is observed at ∼210–245 cm^−1^(34). We could not observe this peak due to the detection limits of the device we use. The carboxylate stretching vibration of the cysteine and AgC molecules were respectively observed at 1575 cm^-1^ and 1578 cm^-1 ^(33). The hydroxyl bending vibrations, with carboxylic acid, the cysteine and AgC molecules were observed at 1393 cm^-1^ and 1400 cm^-1^. A peptide bond is an amide-type of the covalent chemical bond, and it is one of the best indicators of protein presence. The peak of amide for the TRAIL protein (C=O stretching) was observed at 1640 cm^-1^ for AgCTP molecule (Figure 2b) (35). However, the ether stretching vibration for PEG was observed at 1086 cm^-1^ (36, 37).

Particle sizes of AgNPs were found as 41.21 ± 3.80 nm (AgC), 91.22 ± 7.34 nm (AgCT) and 128.12 ± 8.02 nm (AgCTP) (Figure 2c). However, zeta potential values of AgNPs were measured -19.22 ± 3.07 mV (AgC), -27.3 ± 4.34 mV (AgCT) and -28.75 ± 2.02 mV (AgCTP) (Figure 2d).

*Cell viability, proliferation and hemocompatibility with AgNPs*

**Figure 3:**
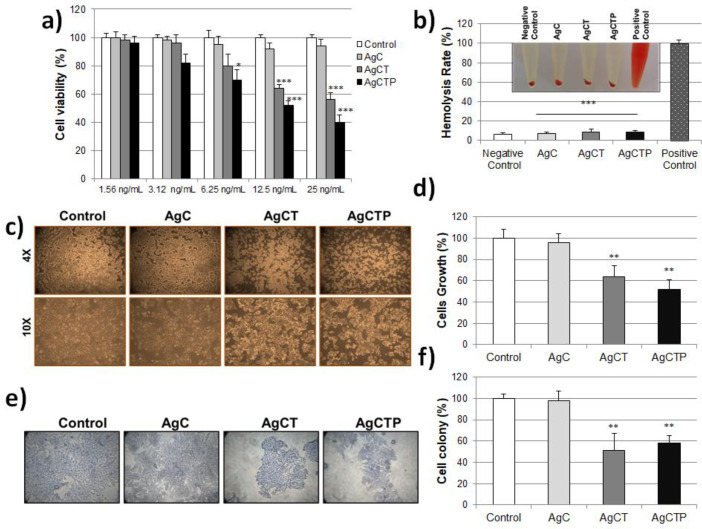
(a) Cell viability measurement with MTT assay after AgCT NPs treatment (1.56–25 ng/mL) of HT-29 cell lines for 24 h. (b) *In-vitro* hemolysis assay of AgNPs in red blood cells. The ratio of hemolysis in red blood cells with treated AgCT NPs ^(***^p ˂ 0.001 compare to positive control). Effect of AgCT NPs (25 ng/mL) on the cell survival in HT-29 human colon cancer cells for 24 h (c) Cell morphological changes (d) Ratio of cell growing ^(**^p ˂ 0.01 compare to control) (e) Cell colony formation stained with crystal violet (f) Ratio of cell colony ^(**^p ˂ 0.01 compare to control) (Results are representative of three biological replicates).

 HT-29 cells were treated with AgNPs at different concentrations (1.56-25 ng/mL) for 24 h, and cytotoxicity analysis was determined using MTT assay. Figure 3a shows, AgC did not produce a significant reduction in viability of HT-29 cells. However, AgCT up to 12.5 ng/mL and AgCTP up to 6.25 ng/mL did not significantly reduce the viability of HT-29 cells. Especially, this reduction was 2.5 fold in AgCTP at 25 ng/mL compared to control. Moreover, AgCTP NPs generally showed more decrease in cell viability than AgCT NPs. 

The behavior of AgNPs under physiological conditions, with a non-toxic effect, was promising for *in-vivo *applications. Figure 3b shows the hemolysis rate for the AgNPs (25 ng/mL). The results have shown that the hemolysis rates for the AgNPs were statistically significant according to positive controls and were almost equivalent to negative controls.

It was possible to associate further decline with the EPR effect. Because the total cell count significantly reduced after 24 h treatment with 25 ng/mL AgCT and AgCTP NPs, microscopic evaluations for morphological changes were also made (Figure 3c). AgCTP NPs treated cells showed loss of membrane integrity, cell growth inhibition, cytoplasmic condensation, creating curved forms, and apoptosis body. The control cells showed cell-cell interaction via normal morphological alterations. The concentration of 25 ng/mL AgCT and AgCTP NPs showed an antiproliferative effect on the proliferation of colon cancer cells compared to control (Figure 3d).

*The colony-forming ability of HT-29 cells with AgNPs treatment*

The colony-forming ability of HT-29 cells was evaluated in cell culture by treatment of AgNPs. Twenty-five ng/mL AgCT or AgCTP NPs showed a significant decrease in the number of colon cancer cell colonies compared to control. However, there was no significant reduction in treatment with 25 ng/mL AgC compared to control. These data have shown that AgCT and AgCTP NPs diminished the metastatic effect by carrying out TRAIL proteins on HT-29 colon cancer cells (Figures 3e-3f). 

*Cell apoptosis with AgNPs*

**Figure 4:**
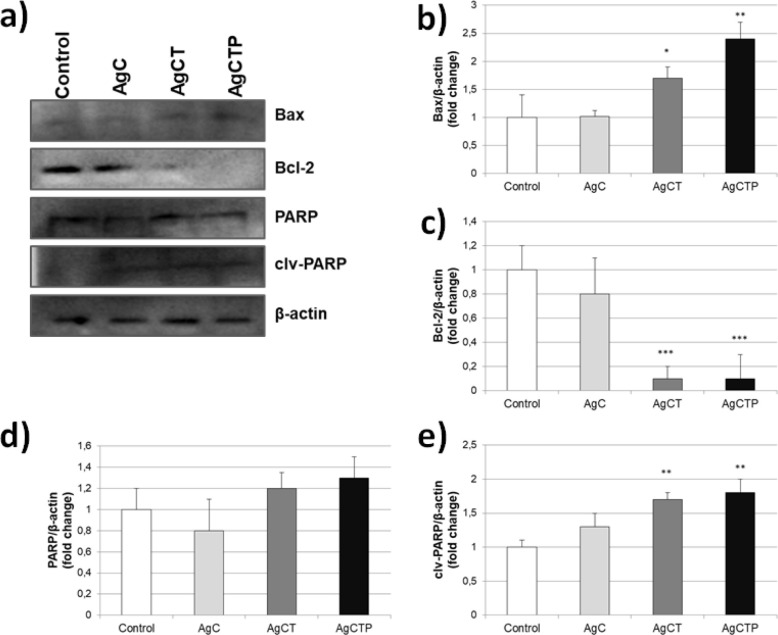
Effects of AgCT NPs (25 ng/mL) on apoptotic and anti-apoptotic proteins in HT-29 human colon cancer cells for 24 h. (a) Western blot bands of expression levels of Bax, Bcl-2 and PARP proteins. Each protein band was normalized to the intensity of β-actin used. Western blot densitometry analysis of (b) Bax (c) Bcl-2 (d) PARP (e) clv-PARP protein expression levels. (^*^p ˂ 0.05, ^**^p ˂ 0.01, ^***^p ˂ 0.001 compare to control cells, (Results are representative of three biological replicates).

To investigate the effects of AgNPs on apoptosis in HT-29 cell line, these cells were treated with 25 ng/mL AgNPs for 24 h and then the expression levels of Bax, Bcl-2, PARP, clv-PARP and β-actin (control) proteins were assessed by western blot. Our results showed that AgC NPs had no significant effect on apoptosis (Figures 4a-4e). However, AgCT and AgCTP NPs showed statistically significant differences in Bax, Bcl-2 and clv-PARP expression levels except PARP proteins involved in apoptosis regulation.

## Discussion

The functionalization of AgNPs with therapeutic increases efficacy in targeted drug delivery in cancer, improving the efficacy of nanomedicine-based theranostics applications. Today, AgNPs play a special role in cancer therapy because of their unique physical and chemical properties.

AgNPs surface layer may be functionalized with various chemical functional groups or moieties to enhance binding drug or proteins. Surface functionalization of silver nanoparticles can be performed by cysteine that changes some characteristic features of silver nanoparticles. Most publications have reported that the characteristic peak around 400-450 nm seen in the UV-vis spectrum is specific for AgNPs (38, 39). As cysteine was not used as a functional group in these publications, it was normal to observe characteristic peaks around 400-450 nm for AgNPs. But there are also publications similar to our results indicating that the characteristic peak was about 390 nm. In these publications, cysteine was used as a functional group to form AgNPs (34, 40-42).

The particle size of the NPs is an important factor affecting the delivery of therapeutic agents to solid tumors. It has been reported that NPs larger than 500 nm are rapidly cleared from the body by the macrophage and monocytes of RES while particles smaller than 10 nm can undergo renal clearance; larger than 200 nm can experience splenic clearance (43,44). Therefore, it is possible to say that NPs less than 200 nm are the most suitable for intravenous applications (45). However, 341.6 nm-sized TRAIL/Dox HSA-NPs have been shown to be effective for lung cancer *in-vitro* and *in-vivo *by aerosolization (46). If we look at the size of synthesized AgC, AgCT and AgCTP NPs, they were 41 nm, 91 nm and 128 nm, respectively. Since additional groups were added to the molecule, particle size increased. For example, spherical structures of TRAIL NPs and TRAIL-PEG NPs, with heparin and poly-L-lysine, were stated to have a size of 205.4 and 213.3 nm, respectively. Despite this size, TRAIL-PEG NPS was stable in circulation for a long time compared to TRAIL (47). In addition, NPs with zeta potential value greater than ± 30 mV as numeric are considered highly stable in the dispersion medium (48). Thus, our synthesized AgCTP NPs may remain stable *in-vitro* and/or *in-vivo *for a long time.

TRAIL is a safe, powerful and well-tolerated anti-cancer agent. It can induce apoptosis by using an extrinsic pathway via the proapoptotic death receptors (DR4 and DR5) in cancer cells but not in normal cells (49). Therefore, it has also been used in many clinical trials as well as *in-vitro* studies (50-54). However, TRAIL use in the clinic is not suitable because of its short biological half-life, which was started approximately 0.5 to 1 h (51). Because the amount of dose that TRAIL is effective cannot be delivered into the tumor tissue. Therefore, TRAIL has commonly used in combination with other anti-cancer agents to create a synergistic effect (55-60). Whereas the power of TRAIL can be revealed by coating with materials such as PEG (53, 61 and 62) and/or by functioning with NPs (26, 63 and 64) without the need for combined treatments. For example, Kim and *et al.* have shown that PEGylated TRAIL molecules have long-term activity proportional to the molecular weight of PEG (61). Lim *et al.* have emphasized that TRAIL-PEG-NPs formulation extended the half-life of TRAIL by 28.3 fold in rats (62). In the xenograft mouse model with a similar strategy, it was observed that serum half-life of the human serum albumin (HSA)-TRAIL increased 15 fold compared to rTRAIL (65). By Bae and *et al.,* TRAIL/Transferrin/Dox HSA-NPs were found to be retained in circulation for 32 h (66). Additionally, it is stated that TRAIL-loaded poly(lactic-co-glycolic acid) microspheres showed sustained TRAIL release for up to 10 days *in-vivo *xenograft tumor model (67). The main purpose of synthesized AgCT NPs coating with PEG was to prolong the half-life of AgCT NPs. Furthermore, PEGylation improved targeting to cancerous cells by the EPR effect and would prevent AgCT NPs from interacting with other molecules in circulation.

In studies conducted with TRAIL, it has been observed that Bcl-2 protein is generally overexpressed due to the non-effective dose of anti-cancer agent and thus improves resistance to apoptosis. In a study by Rudner and et al., it has been shown that TRAIL up to 10 ng/mL increased expression of Bcl-2 despite tetracycline-repressed Bcl-2 (68). Meanwhile, HT-29 cancer cells underwent apoptosis by increased Bax expression and loss of Bcl-2 after treatment with AgCTP NPs (25 ng/mL). Increased Bax level is a clear sign of apoptosis, while decreased Bcl-2 level indicates no resistance to apoptosis.

As a result, we suggest that cysteine, PEG and TRAIL conjugated AgCTP NPs can be used as a safe and effective anti-cancer agent. Considering hemolysis assay results, this conjugation can also be used *in-vivo *studies without creating a toxic effect.

## Supplementary Materials


