# Supplementary material for: TRAIL Conjugated Silver Nanoparticle Synthesis, Characterization and Therapeutic Effects on HT-29 Colon Cancer Cells
Source: Iran J Pharm Res. 2021 Spring;20(2):45–56. doi: 10.22037/ijpr.2020.112069.13514 (PMC8457744; doi:10.22037/ijpr.2020.112069.13514)
Supplement: Supplementary file 1 [file ijpr-20-45-s001.pdf]

Supplementary Materials for

TRAIL Conjugated Silver Nanoparticle Synthesis, Characterization and Therapeutic Effects on HT-29 Colon Cancer Cells

Fatih Birtekocak[a], Gulen Melike Demirbolat[b] and Ozge Cevik[a] *

*To whom correspondence should be addressed. E-mail: ozge.cevik@adu.edu.tr

Volume 20, Issue 2 (Spring 2021)

This PDF file includes:
Figure S1

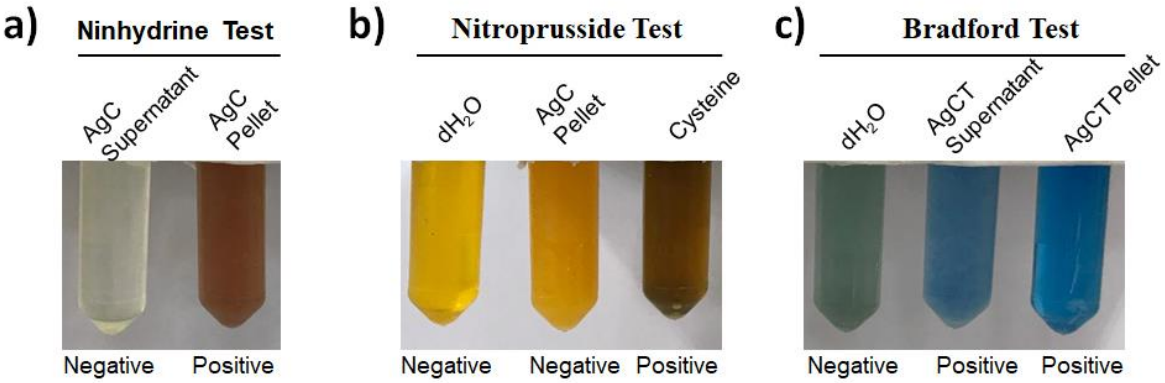

Figure S1. Qualitative validation tests for the verification of syntheses (a) Ninhydrin test (b) Nitroprusside test (c) Bradford test.
